# State Licensure for the Practice of Medicine

**Published:** 1894-08

**Authors:** 


BUFFALO MEDICAL AND SURGICAL JOURNAL
A MONTHLY REVIEW OF MEDICINE AND SURGERY.

*EDITORS:*

THOMAS LOTHROP, M. D. - - WM. WARREN POTTER, M. D.

All communications, whether of a literary or business nature, should be addressed to the managing editor: 284 Franklin Street, Buffalo, N. Y.

Vol. XXXIV. AUGUST, 1894. No. 1.

### STATE LICENSURE FOR THE PRACTICE OF MEDICINE.

The completion of another academic year of state licensure for the practice of medicine in New York, affords a fitting opportunity for offering remarks relating to the year's work, as well as observations on the principle involved and the relationship it bears to other states.

Though the present medical practice act has been on the statute books for three years, it was, as is well known, rendered practically nugatory for the first two years of its existence by reason of the passage of exemption laws, that covered the major portion of the graduates who would otherwise have come up for state examination. For the first year these exemptions were practically the whole number of students, nine hundred and thirteen having filed exemption papers. Less than one hundred applied for state license, and most of these were graduates of medical colleges in other states or foreign countries. The second year made a better showing, as exemptions were fewer. During this period the state examining and licensing board, representing the Medical Society of the State of New York, passed two hundred and forty-four candidates and rejected twenty.

We now come to the consideration of the operations of the law during the third year of its life, and here we shall observe that exemptions have nearly ceased. This is, however, not absolutely the case, for, strange to say, all sorts of flimsy reasons are trumped up to avoid the state examination, and this in spite of the fact that the state license is a far more valuable instrument than the diploma of any medical school, and is worth all the effort that it costs in time and money. It enables a man to practise anywhere in the United States, and even foreign countries are beginning to accord it recognition. It bears the stamp of the great Empire State, and is a commission that ought to beget pride in any man who holds it. It should be remarked, however, in all fairness, that some men, recognizing the value of the state license, have taken the examination and obtained it, when, under a strict construction of the law, they could not be compelled to do so. All honor to these men, say we! They stand out in bold relief against that class of men who have stretched a point to obtain exemption.

In this third academic year, the examinations by the state board have reached nearly four hundred in number, but the percentage of rejections has been considerably less. The answer papers have shown much more careful training on the part of the schools. There is still, however, great room for literary improvement, and the colleges will find it important to scrutinize carefully students' preliminary training at the entrance examination. Very often a candidate shows excellent medical proficiency and a knowledge of his subject, but makes a bad-looking paper by reason of deficient preliminary education. How grotesque it would appear to one of our English medical friends if he should walk into the office of one of the licensees of the State of New York only to find him speaking poor English and writing it worse. Yet this thing is very likely to happen unless the medical examining and licensing board guards the portals to state licensure with a jealous care in this respect.

The preliminary requirements are yet inadequate or are enforced with too great laxity to prevent incongruities like that above cited, hence the necessity of all the greater care on the part of the examiners.

At the last meeting of the Association of American Medical Colleges it was resolved to require four years of college teaching, each to be of six months' duration, as a requirement for graduation. Under the medical practice act of New York, the college courses were raised to three years as a minimum, and if no other reform had been inaugurated by the law, this alone would have been worth all the time and labor and patience expended in obtaining its enactment. Some of the colleges were very much disturbed at this proposed increase of the time for college teaching, and ostensibly based their opposition to the law on that ground. But the adoption of a four years' requirement by the American Association of Medical Colleges will necessitate the amendment of the

New York law to meet this increase. The people are becoming aroused on the subject of higher medical education, and will be intolerant of medical schools whose "learned professors" stand in the path of this ever-moving procession.

Canada has increased the terms of her college courses to five years, and we have no doubt that the time will soon come when this will be required in the United States. We have noticed that already one Western college, the St. Louis College of Physicians and Surgeons, has announced its intention to earnestly advocate this increase.

It is to be regretted that the great State of Ohio failed to enact the law before its last legislature, providing for separate state examinations and license. Kansas, too, is in close sympathy with Ohio in the failure to establish such a law. Another year, however, will probably place both of these great commonwealths in the line of improved medical education with better practice acts than they would have had, had they succeeded at first.

The New York law needs two amendments to bring it abreast of the spirit of the age. One of these should provide for a higher standard of preliminary education and more rigid entrance examinations, and the other should demand a four years' course and make it obligatory for the colleges to require this, and for these amendments we shall ever work and pray.

